# Our New York Letter

**Published:** 1887-12

**Authors:** 


### Correspondence.

### OUR NEW YORK LETTER.

INVESTIGATION OF ALLEGED ABUSES OF THE INSANE IN NEW YORK—A NEWSPAPER TRICK—DEATH OF DR. JAMES KNIGHT AND OF DR. JOHN M. CARNOCHAN—PROMOTION OF DR. V. P. GIBNEY—ANTIPYRIN AND ANTIFEBRIN IN DISEASES OF THE NERVOUS SYSTEM.

*To the Atlanta Medical and Surgical Journal:*

Some months ago considerable excitement was aroused in regard to numerous alleged abuses which were said to exist in our public city insane asylums, and an investigating committee, appointed for the purpose, spent a number of weeks in trying to ascertain what truth there was in the statements of patients and their friends. About this time an enterprising newspaper conceived the idea of employing a young woman who would feign insanity, gain admission to the female lunatic asylum, and procure information for the newspaper. A young female journalist was selected for this purpose and she began her duties by going to a working woman's boarding-house with but seventy cents in her possession and applying for board. Here she was told there was no room for her, but after prevailing on the landlady for sometime and declaring her willingness to sleep on the floor, she was finally taken in. The same evening, while sitting in the parlor with the other boarders, she told a long story of her lost trunks, and soon began to cause a sensation by her strange conversation. Upon going to her room for the night she stood before the mirror pulling her hair and talking to herself, until her room-mate became frightened and alarmed the household.

The other boarders all expressed their sympathy for her, and one bolder than the rest declared that the girl had had some unfortunate love affair, which was affecting her mind, and volunteered to watch her during the night. On the following morning a police officer was sent for to remove the troublesome boarder, and she was taken before a judge of one of our city courts, who, after talking to her awhile, decided that she was insane, and had an ambulance telegraphed for. The ambulance surgeon examined her eyes, and after asking a few questions, she was taken to Bellevue Hospital and placed in the insane pavilion, where she remained for five days. During this time she was examined by four or five experts, who had no hesitation in pronouncing her insane, and it was finally decided to transfer her to the Female Lunatic Asylum on Blackwell's Island. She remained ten days in this institution, and during this period continued to deceive the doctors by her strange actions. At the end of this time the secret was purposely let out and she was released.

Although the journal for whom all this was done claims that its object was to investigate the asylum abuses and prove how little doctors know about insanity, yet the real purpose probably was to benefit the newspaper, and nothing new appears to have been proved so far.

Since my last letter two distinguished members of our profession, Dr. James Knight and Dr. John Murray Carnochan, have dropped from the ranks. Dr. Knight will be remembered by many as the chief surgeon of the Hospital for Ruptured and Crippled at 42d street, and Lexington avenue, with which institution he has been connected ever since its origin. Dr. Knight was a member of many prominent medical organizations and the author of several works on orthopedic subjects. He died in his seventy-seventh year, and the vacancy thus left in the hospital will be filled by Dr. V. P. Gibney. Dr. Carnochan died of apoplexy, in his seventy-first year, after an eventful and successful life. He was instructed in early life for a physician by the best teachers of the times in France and also America, and soon after beginning practice in New York was appointed to take charge of the Hospital for Emigrants on Ward's Island. As a surgeon he was eminently successful, and soon after this first appointment he was selected to fill the chair of surgery in the Medical College of the University of New York. During the administration of John T. Hoffman as Governor of the State of New York, Dr. Carnochan was appointed health officer of this port, which position he filled for two years. As a writer on surgery he gained considerable reputation, and a series, entitled "Contributions to Operative Surgery," was still unfinished at the time of his death.

At a recent meeting of the Neurological Society the subject of the "Use of Antipyrin and Antifebrin" was discussed.

Dr. T. S. Robertson stated that he had been pleased with the effects of antipyrin in inflammatory rheumatism and had seen it reduce fever and control the pain. He had also used it in over 100 cases of cephalalgia and neuralgic troubles with relief in 90 per cent. of the patients. In the pains of locomotor ataxia it was useful and he considered it better than chloral in sleeplessness. Dr. W. A. Hammond's experience with antipyrin had been entirely negative and unsatisfactory. He had used it and also antifebrin in neuralgias, insomnia, and in all kinds of headaches, giving gr. xv t. i. d. He considered a hypodermic of morphine far superior to shorten an attack of migraine. Dr. Sachs was a firm believer in the use of antipyrin for migraine and he had given it frequently with relief in 20 minutes. In insomnia he considered it a true narcotic and it was not unusual for a patient to sleep 9 to 10 hours after a gr. xx dose. The chairman, Dr. Dana, was very much surprised with Dr. Hammond's remarks, and suggested that he ought to watch his clinical assistants closer. One of these assistants he said had been cured of a severe migraine by antipyrin and at present prescribed it often at Dr. Hammond's Post Graduate School Clinic. He believed that antifebrin was a spinal depressant and consequently might be useful in epilepsy. In one case he had seen it act beneficially. In closing the discussion Dr. Robertson stated that he had found antipyrin, dissolved well in Vichy and a dose of 20 or 30 grains, could be given with safety.

*New York, November, 1887.*

